# Antimicrobial Peptides in 2014

**DOI:** 10.3390/ph8010123

**Published:** 2015-03-23

**Authors:** Guangshun Wang, Biswajit Mishra, Kyle Lau, Tamara Lushnikova, Radha Golla, Xiuqing Wang

**Affiliations:** 1 Department of Pathology and Microbiology, University of Nebraska Medical Center, 986495 Nebraska Medical Center, Omaha, NE 68198-6495, USA; 2 Institute of Clinical Laboratory, Ningxia Medical University, Yinchuan 750004, China

**Keywords:** antimicrobial peptide, bacterial detection, biofilms, mechanism of action, nanoparticle, peptide discovery, sensors, structure-based design, surface coating

## Abstract

This article highlights new members, novel mechanisms of action, new functions, and interesting applications of antimicrobial peptides reported in 2014. As of December 2014, over 100 new peptides were registered into the Antimicrobial Peptide Database, increasing the total number of entries to 2493. Unique antimicrobial peptides have been identified from marine bacteria, fungi, and plants. Environmental conditions clearly influence peptide activity or function. Human α-defensin HD-6 is only antimicrobial under reduced conditions. The pH-dependent oligomerization of human cathelicidin LL-37 is linked to double-stranded RNA delivery to endosomes, where the acidic pH triggers the dissociation of the peptide aggregate to release its cargo. Proline-rich peptides, previously known to bind to heat shock proteins, are shown to inhibit protein synthesis. A model antimicrobial peptide is demonstrated to have multiple hits on bacteria, including surface protein delocalization. While cell surface modification to decrease cationic peptide binding is a recognized resistance mechanism for pathogenic bacteria, it is also used as a survival strategy for commensal bacteria. The year 2014 also witnessed continued efforts in exploiting potential applications of antimicrobial peptides. We highlight 3D structure-based design of peptide antimicrobials and vaccines, surface coating, delivery systems, and microbial detection devices involving antimicrobial peptides. The 2014 results also support that combination therapy is preferred over monotherapy in treating biofilms.

## 1. Introduction

Antimicrobial peptides, or host defense peptides, are important components of innate immune systems. This field is currently moving rapidly. On one hand, there is an urgent demand for novel antimicrobials due to the current trend of reduction in the potency of commonly used antibiotics. On the other hand, our research now pays more attention to innate immune systems where antimicrobial peptides play an essential role. An imbalanced expression of antimicrobial peptides has been implicated in human disease [1,2,3,4,5]. All these research activities around the world led to a substantial increase in the number of scientific papers on antimicrobial peptides. In 2014 alone, a search of the PubMed using “antimicrobial peptides and 2014” returned 7562 publications (~20 articles per day) [6]. About 10% of these publications are review articles. However, a summary in the style of annual report is lacking. During our regular update of the Antimicrobial Peptide Database (APD) (*http://aps.unmc.edu/AP*) [7,8,9] in the past years, we noticed new peptides of outstanding interest and created a website for them (*http://aps.unmc.edu/AP/timeline.php*) [10]. We also felt the need to write a story on these interesting molecules. Since the antimicrobial peptide field is rather broad, a detailed report on every aspect of the research is out of the scope of this review. Instead, we chose to highlight the antimicrobial peptide research by focusing on the following topics: (1) Of the new peptides discovered in 2014, are there any unique members that expand our current knowledge of natural antimicrobial peptides? (2) For known peptides, have we uncovered new functions that fill in our knowledge gap? (3) What progress have we made in mechanistic studies? Have any of our existing views been challenged? What about the genetic basis of bacterial resistance? (4) Are there advances in peptide applications? In the following, we discuss these four aspects of the antimicrobial peptide research based on the *new* discoveries made during 2014. We apologize if your important work did not fit into the scope of this article or escaped our attention.

## 2. New Host Defense Peptides Reported in 2014

This section features new antimicrobial peptides discovered in 2014. Two major methods were utilized for peptide discovery: a combination of chromatographic approaches [11,12,13,14,15,16,17,18,19,20,21] and genomic and proteomic approaches [22,23,24,25]. The proteomic approach has the potential of identifying a large number of peptides. However, we only registered peptides into the APD database if they have a known amino acid sequence (usually less than 100 amino acids) and demonstrated antimicrobial activity. In 2014, 104 new antimicrobial peptides were registered in the APD [7,8]. This 2014 total is comparable to those annual totals of peptides (over 100) collected into the APD since 2000 [26]. In the following, we highlight unique peptides from various life kingdoms.

Of the 104 new antimicrobial peptides, 29 bacteriocins (*i.e.*, bacterial antimicrobial peptides) were isolated from the bacterial kingdom. The peptide BacFL31 is unusual in that its N-terminal amino acid sequence contains six hydroxyprolines (X in the sequence GLEESXGHXGQXGPXGPXGAXGP) [11]. Baceridin, a non-ribosomally synthesized circular peptide with only six amino acids (50% d-amino acids), was isolated from a plant-associated Bacillus strain [12]. This peptide can inhibit cell cycle progression and causes apoptosis in cancer cells independent of p53. It is the most hydrophobic peptide (100%) and the shortest circular peptide in the APD (Table 1). Lassomycin was found to be similar to lassos since its aspartic acid 8 forms a bond with the N-terminal glycine. This peptide kills *Mycobacterium tuberculosis* by binding to ATP-dependent protease ClpC1P1P2 [13]. It is exciting that humans have reached organisms deep in the sea. Using transformation-associated recombination (TAR) technology, Yamanaka *et al.* succeeded in cloning and expression of a silent lipopeptide biosynthetic gene cluster from the marine actinomycete *Saccharomonospora* sp. CNQ-490 to produce taromycin A, a daptomycin analog [14]. In addition, several lipopeptides were found from a marine bacterium *Bacillus subtilis*. One of them, gageotetrin A, consists of only leucine and glutamic acid followed by a new fatty acid 3-hydroxy-11-methyltridecanoic acid at the C-terminus [27]. Interestingly, these peptides displayed rather good antibacterial activity (0.01–0.06 µM) against *Staphylococcus aureus*, *B. subtilis*, *Salmonella typhimurium*, *Pseudomonas aeruginosa*, *Rhizoctonia solani*, *Colletotrichum acutatum*, and *Botrytis cinera*. Although gageotetrin A is a conjugate, it possesses the shortest peptide sequence (Table 1) in the APD. The structure of anionic gageotetrin A (peptide + fatty acid) is clearly different from synthetic ultra-short lipopeptides (fatty acid + peptide), which are usually cationic to mimic cationic antimicrobial peptides [28]. Gageotetrin A has a simpler molecular design compared to anionic daptomycin, the first lipopeptide antimicrobial approved by FDA in 2003 [29]. Another peptide, sonorensin with broad activity spectrum against both Gram-positive and Gram-negative bacteria, was also identified from a marine bacterium *Bacillus sonorensis* MT93. It possesses a unique amino acid sequence with multiple copies of the CWSCXGHS motif, where X is methionine or alanine (Table 1). Sonorensin is the first characterized bacteriocin from the heterocycloanthracin subfamily [30]. These successful examples prove that it is likely to identify novel antimicrobial peptides from unexplored organisms.

The discovery of new antimicrobial peptides from the fungal kingdom is lagging behind other life kingdoms. In 2014, we collected only one defensin-like peptide from *Coprinopsis cinerea*. Like fungal plectasin and eurocin [31,32], copsin inhibited cell wall synthesis by binding to lipid II [33]. These fungal peptides share the same 3D fold, comprising one α-helix packed with a two-stranded β-sheet. Differing from plectasin and eurocin with three disulfide bonds, however, copsin is stabilized by six disulfide bonds. In addition, the N-terminus of copsin is modified into a pyroglutamate (17 peptides in the APD with such a modification [8]), furthering conferring stability to the peptide against high temperatures and protease digestion. Thus, fungi constitute yet another important kingdom for novel antimicrobial discovery.

In 2014, five new antimicrobial peptides were characterized from the plant kingdom and one of them is quite unique. Different from many disulfide bond stabilized defensins with a β-sheet structure, EcAMP3 is a disulfide-stabilized hairpin-like α-helical peptide. It is the first such peptide that inhibits phytopathogenic bacteria [20]. Hispidalin from winter melon *Benincasa hispida* [34] shows only 31% sequence similarity to tachycitin from horseshoe crabs [35] and amphibian brevinin-1PRb [36] based on sequence alignment in the APD [7]. Since hispidalin is a newly discovered peptide, it has not been trained in the existing programs. Not surprising, several online machine-learning programs were unable to predict it as an antimicrobial peptide [37,38,39].

**Table 1:** Select antimicrobial peptides discovered in 2014.

APD ID	Name	Source	Peptide amino acid sequence	Unique features ^1^
2381	Gageotetrin A	Bacteria	LE	The shortest lipopeptide
2397	Sonorensin	Bacteria	CWSCMGHSCWSCMGHSCWSCAGHSCWSCMGHSCWSCMGHSCWSCAGHCCGSCWHGGM	Repeating CWSCXGHS motif
2372	Baceridin	Bacteria	WAIVLL	The shortest circular peptide consisting entirely of hydrophobic amino acids
2440	Copsin	Fungi	QNCPTRRGLCVTSGLTACRNHCRSCHRGDVGCVRCSNAQCTGFLGTTCTCINPCPRC	The first fungal defensin with six disulfide bonds
2407	Hispidalin	Plants	SDYLNNNPLFPRYDIGNVELSTAYRSFANQKAPGRLNQNWALTADYTYR	A unique peptide with 31% similarity to known sequences. Not predicted by existing programs
2477	EcAMP3	Plants	GADRCRERCERRHRGDWQGKQRCLMECRRREQEED	The first disulfide-stabilized hairpin-like helical peptide that inhibits phytopathogenic bacteria
2424	Crotalicidin	Animals	KRFKKFFKKVKKSVKKRLKKIFKKPMVIGVTIPF	Rich in lysine (38%)

^1^ Additional peptide properties can be found in the APD database (*http://aps.unmc.edu/AP*) [8] using peptide ID in the table. A full list of the 2014 antimicrobial peptides can also be studied there.

Of the 104 antimicrobial peptides found in 2014, 69 originated from animals. This is consistent with the overall picture in the APD that antimicrobial peptides from the animal kingdom dominate [26]. Moreover, amphibians remain a major source for discovering natural antimicrobial peptides, accounting for 35% of the 2014 total (38.8% of the entire database entries). Most of these new sequences resemble the known frog antimicrobial peptides, which are linear and have the potential to form a helical structure [18,19]. Although cathelicidins have been identified from a variety of animals, ranging from birds, fish, and reptiles, to mammals [40], candidates from amphibians were not reported until 2012 [41]. In 2014, two new members appeared [42], leading to a total of six amphibian cathelicidins in the APD (five helical and one glycine-rich). These cathelicidins are quite distinct from the main body of amphibian peptides. For example, cathlicidin RC-1 has a high content of lysines (32%). Crotalicidin [17], a homologous snake cathelicidin, contains an even higher content of lysines (38%) (Table 1).

Also in 2014, some known human peptides or proteins were demonstrated to be antimicrobial. These include human α-defensin 6 (HD-6), β-defensin 120 (DEFB120), chemokine CCL24 (eotaxin-2), CCL26 (eotaxin-3), and human ribonuclease 6 (RNase 6). While HD-6 is active against *Bifidobacterium* *adolescentis* [43], recombinant DEFB120 is active against *Escherichia coli*, *S. aureus*, and *Candida albicans* [44]. Eotaxin-1 (CCL11), eotaxin-2, and eotaxin-3 are known chemokines, which are also active against the airway pathogens *Streptococcus pneumoniae*, *S. aureus*, *Haemophilus influenzae*, and *P. aeruginosa* [45]. In addition, human RNase 6 is inducible and shows activity against uropathogens, underscoring its defense role in the urinary tract [46]. These characterized members further expand the known reservoir of human host defense peptides and proteins reviewed in 2014 [47].

## 3. New Light on Known Human Antimicrobial Peptides

Antimicrobial peptides may be constitutively expressed to keep defined loci in a healthy state [1,48]. Compared to neonatal and adult keratinocytes, the corresponding fetal cells express much more human antimicrobial peptides for host defense [49]. Alternatively, these molecules can also be induced upon bacterial invasion. For example, a human cathelicidin peptide is induced in skin fat cells upon *S. aureus* infection, underscoring the significance of adipocytes in host defense [50]. Previously, Gallo and colleagues also found that overexpression of cathelicidin PR-39 protected animals from group A Streptococcus (GAS) infection [51]. Interestingly, the gut possesses both constitutively expressed and induced antimicrobial peptides. While human cathelicidin LL-37 and β-defensins 2-4 (hBD-2 to HBD-4) are induced, human α-defensin 5 (HD-5), HD-6, and β-defensin 1 (hBD-1) are constitutively expressed [52]. These constitutively expressed human peptides also play a special role in host defense. In 2014, HD-5 was shown to be especially potent against the most virulent form of *Clostridium difficile*, thereby preventing its infection of small intestine. This is a significant observation considering that *C. difficile* can evade the action of other host microbicidal peptides and disturb the balance of gut microbiota [53]. Human papillomavirus (HPV) infections can lead to cervical cancer and HD-5 can prevent viral entry [54]. In addition, Wiens and Smith showed that HD-5 directly interferes with a critical host-mediated viral processing step, furin cleavage of L2, at the cell surface [55]. Structurally, HD-5 can form a disulfide bond swapped dimer *in vitro* [56]. It should be interesting to test whether this dimer is linked to host defense *in vivo*.

How HD-6 plays the defense role in human gut has been puzzling for years. Similar to human hBD-1 [57], Schroeder *et al.* found that human Paneth cell HD-6 only exerted antibacterial activity under reduced conditions, establishing it as a bona fide antimicrobial peptide [43]. This reduction may be achieved *in vivo* by the NADPH thioredoxin-reductase system. *In vitro*, removal of the N-terminal two amino acid residues of HD-6 enabled a full reduction by dithiothreitol without influencing its activity. Such a truncated form was isolated previously from ileal neobladder urine [58]. In addition, HD-6 can form neutrophil extracellular traps (NETs) to trap invading microbes [59]. Thus, recent breakthroughs have uncovered two possible mechanisms for HD-6 in host defense.

The importance of environmental conditions for antimicrobial activity is not limited to α-defensins. In 2014, Abou Alaiwa *et al.* showed that the composition of the airway surface liquid is critical for human LL-37 and hBD-3 to kill inhaled and aspirated bacteria. A decrease in pH from 8 to 6.8 in pulmonary airway reduced the activity of both peptides against *S. aureus* as well as synergistic effects between innate immune peptides [60].

It is known that pH modulates the oligomerization state of human LL-37. At acidic pH, LL-37 is monomeric; it aggregates at physiological pH [61]. The mode of oligomerization was also studied in 2014 by using disulfide-linked dimers [62]. NMR studies confirmed this pH-dependent phenomenon [63]. However, the link of this phenomenon to biology was not clear. In 2014, Gao and colleagues reported that LL-37 enhancement of signal transduction by Toll-like receptor 3 (TLR3) is regulated by pH [64]. Upon acidification in endosomes, oligomerized LL-37 dissociates, allowing the release of delivered dsRNA to act as an agonist for TLR3 signaling. In contrast, LL-29, a natural fragment of human LL-37 that lacks the C-terminal portion [65], is unable to do so. Since our previous NMR studies found that nearly all the residues of LL-37 (residues 1-36) are involved in oligomerization [63,66], the C-terminal portion of LL-37 might be involved in the tetramer formation of LL-37. The salt bridges, likely involving R34 and/or E36, can be disrupted at acidic pH, providing a molecular basis for pH-dependent oligomer dissociation and dsRNA release.

All these examples above underscore that environmental conditions are an important mediator of the function of antimicrobial peptides. There are also other mediators that regulate peptide activity, including proteases, metals, salts, and chemical modifications. While 3D triple-resonance NMR studies show that the helical region (residues 2-31) of LL-37 is responsible for both membrane and lipopolysaccharides (LPS) binding [63], citrullination of arginines can reduce its ability in preventing endotoxin-induced sepsis [67]. Likewise, ADP-ribosylation of four out of the five arginines of human LL-37 may regulate this property [68]. A more recent discovery reveals that during *S. aureus* invasion, skin adipocytes can replicate rapidly and produce a cathelicidin peptide longer than LL-37 to prevent infection [50]. Previously, a different form of human cathelicidin, ALL-38, was also isolated from the human reproductive system [69]. Therefore, human proteases at a defined location play an important role in determining the exact molecular form of mature antimicrobial peptides required for host defense [47].

## 4. Mechanisms of Action of Antimicrobial Peptides and Genetic Basis of Bacterial Resistance

### 4.1. Peptide at Work

**Figure 1:**
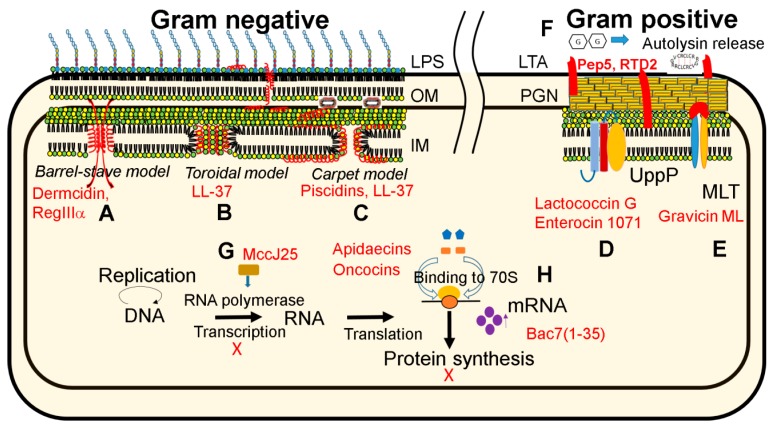
Mechanisms of action of antimicrobial peptides in 2014. Membrane channel formation (**A**) is proposed for dermicidin [76] and transmembrane pore formation for C-type lectin RegIIIα [77]. While human LL-37 [78] may form a toroidal pore (**B**), it started with a carpet model [79] (**C**) where antimicrobial peptides such as piscidins [72] are located on the membrane surface. Receptor mediated binding was observed for Lactococcin G and Enterocin 1071, which bind to UppP, an enzyme involved in cell wall synthesis (**D**) [80]. In addition, Gravicin ML binds to maltose ABC receptors (**E**) [81]. Further, RTD2, as well as lantibiotic Pep5, interacts with membranes causing the release of autolysin (**F**) [82]. Beyond membranes, bacterial MccJ25 could inhibit RNA polymerase (**G**) [83], while apidaecins, oncocins [84] and Bac7 [85] inhibit protein synthesis by binding to ribosomal proteins (**H**). Abbreviations used in the figures are OM, outer membrane; IM, inner membrane; PGN, peptidoglycan; LTA, lipoteichoic acid; MLT, maltose transporter. In addition, refer to the text.

Although there are anionic peptides, natural antimicrobial peptides are usually cationic with an average net charge of +3.2 [7,8]. A leading view is that these cationic peptides target negatively charged bacterial membranes. However, other mechanisms are possible [4,5,47,63]. In the case of membrane targeting, how it damages the membranes remains debatable. A variety of possible membrane-weakening mechanisms have been summarized by Vogel [70] and three of them are depicted in Figure 1. These models are helpful and may inspire the design of new experiments to check their validity. In the carpet model [71], antimicrobial peptides are assumed to locate on the membrane surface. Is the peptide flat? In 2014, solid-state NMR studies of piscidins revealed peptide tilting to achieve an optimal interaction. The extent of tilting depends on both peptide sequence and lipid composition. The glycine at position 13 may be important for peptide plasticity [72]. Numerous peptide examples, including human cathelicidin LL-37 [66], possess a similar glycine that may modulate peptide activity against different bacteria. However, there are only a few examples with a defined pore. Structural determination yielded evidence for channel or pore formation. Gramicidin and alamethicin are two known examples [73,74]. Recently, the crystal structure of human dermcidin implies another possible channel [75,76]. In 2014, a C-type lectin is proposed to form a pore in bacterial membranes based on a combined structural determination by X-ray diffraction with electron microscopy data. In this model, six copies of human RegIIIα assemble into a ring structure with a hole in the center [77].

Human α-defensin HNP1 and β-defensin hBD-3 are known to inhibit cell wall synthesis [86,87]. What about θ-defensins [16]? In 2014, Selsted and colleagues asked this question. In collaboration with Sahl, they found that RTD-2 did not bind to lipid II. Rather, it interacted with bacterial membranes in the presence of glucose. In addition, they detected the release of peptidoglycan lytic enzymes (or autolysins) by *S. aureus*. Interestingly, bacterial lantibiotic Pep5 can work in the same manner [82]. There is precedence for such a similarity. Like defensins, it is common for lantibiotics to inhibit cell wall synthesis by binding to lipid II [88]. In addition, some lantibitics and cyclotides share the same phosphatidylethanolamine (PE) lipid target [89,90]. Such a cyclotide binding to PE-rich membrane rafts is proposed to be responsible for activity against cancer and HIV-1 [91]. The similarity between cyclotides and lantibiotics was initially suggested by their similar amino acid composition plots [63]. The mechanistic similarities between thioether bonded lantibiotics and disulfide bonded defensing-like peptides are remarkable (Table 2), supporting a recent universal peptide classification that groups them into one big class: sidechain-sidechain linked peptides [9].

**Table 2:** Mechanistic similarities between thioether-bonded lantibiotics and disulfide-boned peptides.

Mechanism	Lantibiotic Examples	Disulfide-Linked Examples
Inhibition of cell wall synthesis ^1^	Nisin A, lacticin 3147, mersacidin, bovicin HJ50	HNP1, hBD-3, plectasin, lucifensin, eurocin, copsin
Membrane and autolysin release	Pep5	θ-defensins such as RTD-2
Binding to lipid PE	Duramycins, cinnamycin	Kalata B1, cycloviolacin O_2_

^1^ Selected from the APD [7,8]. A more complete list can be searched in the APD.

For years, proline-rich peptides were proposed to act by binding to heat shock proteins [92]. Two papers published in 2014 challenged this view. Krizsan *et al.* found that insect-derived proline-rich apidaecins and oncocins inhibited bacterial protein translation at the 70S ribosome [84]. Both cationic and hydrophobic amino acids of the peptides were involved in such binding. Interestingly, Mardirossian *et al*. also observed that Bac7(1-35), another proline-rich peptide corresponding to N-terminal 35 residues of bovine cathelicidin Bac7, could accumulate within *E. coli* to a high concentration of 340 µM and inhibits protein synthesis by targeting ribosomal proteins [85]. These studies indicate that the well-documented chaperone DnaK is unlikely the major target for proline-rich peptides. Of note, bacterial lasso peptides such as microcin J25 (MccJ25) can inhibit RNA polymerase [83].

Some antimicrobial peptides can associate with DNA. In 2014, Ghosh *et al.* found that the WW motif of indolicidin is essential for DNA binding. They provided high-resolution structural information for the interaction of indolicidin with duplex DNA [93]. Such a structure can be useful for designing novel antibiotics.

Interestingly, some bacteriocins use receptors as the target. While garvicin ML recognizes a maltose ABC transporter, LsbB uses metallopeptidase as the targets [81]. In 2014, Kjos *et al.* found lactococcin G and enterocin 1071 (two-chain bacteriocins) used UppP as the receptor. UppP is an enzyme involved in cell wall synthesis [80]. Such findings not only enrich our view on the mechanisms of action of antimicrobial peptides, but also open new opportunities for antimicrobial development.

Although it is likely that some antimicrobial peptides mainly utilize one mechanism to inhibit bacteria, a single peptide may also deploy multiple mechanisms, rendering it difficult for pathogens to develop resistance. In 2014, Wenzel *et al.* illustrated this using a model arginine and tryptophan-rich peptide RWRWRW-NH_2_ (C-terminal amidation) [94]. The peptide is primarily membrane targeting (e.g., D and L-forms have same activity) and only a very small population can enter the bacterial cell. As a new mechanism, the authors found that multiple surface proteins could be delocalized by the peptide. While the replacement of cytochrome C hinders bacterial energy metabolism, delocalization of MinD interferes with bacterial replication. Another surface protein, MurG, can also be delocalized, leading to impaired bacterial cell wall synthesis. The authors proposed that such bacterial surface protein delocalization by cationic antimicrobial peptides may be a general mechanism. Our own recent findings may provide additional insight into this protein delocalization. Surface attachment is usually mediated by a short amphipathic sequence, which weakly interacts with bacterial membranes [95]. In contrast, cationic antimicrobial peptides are able to better interact with bacterial membranes since they have a broader hydrophobic surface or higher membrane perturbation potential [96]. Such a membrane-binding difference could be one of the fundamental reasons for surface proteins to be replaced by cationic antimicrobial peptides. In addition, membrane binding of short amphipathic sequences requires anionic lipids [95]. When short cationic peptides cause lipid domain formation [94,97,98], the migration of anionic lipids toward cationic antimicrobial peptides could weaken the attachment of surface proteins to the re-organized membranes, causing protein delocalization and loss of function as demonstrated by Wenzel *et al.* [94].

A single peptide can also possess multiple functions and human cathelicidin LL-37 is a typical example for this [99]. The observation that LL-37 can associate with DNA led to the idea that DNA binding may be part of the bacterial killing mechanism as well. However, Mardirossian *et al.* showed that only 5% of LL-37 inhibited protein synthesis [85]. In agreement, we did not observe a correlation between peptide activity and DNA retardation (Lau, K.; Lushnikova, T.; Wang, G., unpublished results). However, we did observe a correlation between membrane permeation and antimicrobial activity of LL-37 fragments [100]. These results support the existing view that membrane permeation and disruption by the helical region (residues 2-31) is the major mechanism via which LL-37 kills bacteria [78,79,97]. It appears that nucleic acid binding plays a more important role in RNA delivery into endosomes [101] and in stabilizing neutrophil extracellular traps to prevent DNA cleavage [102]. Moreover, human LL-37 can associate with cell receptors to trigger signal transduction [103,104]. Interestingly, LL-37 also modulates innate immunity by promoting macrophages to phagocyte bacteria [105] or influencing neutrophil responses to influenza A virus [106]. In addition, overexpressed LL-37, as an antigen, can be specifically recognized by CD4+ and/or CD8+ T cells in psoriasis [107]. It is clear that the multifunctional roles of human LL-37 are realized by its ability to recognize and interact with different molecular targets and immune cells.

### 4.2. Resistance Genes for Pathogens and Survival Skills for Commensal Bacteria

It has been appreciated that bacteria have been co-evolving with host defense peptides [108]. Some have learned how to avoid the attack by cationic peptides. The major mechanism appears to alter cell envelope charge and composition. In addition, an ATP-binding cassette (ABC) transporter coupled with an adjacent two-component system (TCS) also constitutes a resistance module against antimicrobial peptides [109,110]. Elucidation of the genetic basis of bacterial resistance may be helpful for the design of more potent antibiotics. In the following sections, we highlight progress made in this direction during 2014.

#### 4.2.1. Gram-Positive Bacteria

One can identify the bacterial genes involved in peptide response by two methods: proteome analysis or genome analysis. Using proteome analysis of bacitracin-treated and untreated cells, Gebhard *et al.* identified an ABC transporter EF2050-2049 of *Enterococcus faecalis* that mediates resistance against bacitracin [109]. To validate this, they transferred the resistance and regulatory pathway to *B. subtilis*, leading to bacitracin resistance. Thus, the ABC transporter and the TCS are indeed required for resistance to antimicrobial peptides. Nevertheless, a previous genomic analysis identified two such ABC transporters [110], which were induced by bacitracin [109]. Based on these results, the authors proposed a model for the bacitracin resistance network of *E. faecalis*. The presence of bacitracin is initially detected by the EF2752-51 ABC transporter, which relays this signal to an adjacent TCS (EF0926-27). Activation of the regulatory domain of the TCS leads to an increase in expression of the EF2050-49 ABC transporter that confers resistance to antimicrobial peptides. This two transporters and one TCS network mechanism [109] differs from those single ABC transporter and TCS cases where the transporter senses the peptide and relays this signal to the adjacent TCS that upregulates the same ABC transporter for resistance [110].

The five-component system GraXSR-VraFG of *S. aureus* is well-established as the major sensing and resistance system [111] that reduces the toxic effect of cationic antimicrobial peptides by upregulating genes such as *mprF* and *dltABCD.* While MprF can put lysines on anionic phophatidylglycerols (PGs), DltABCD can modify the cell wall by transferring of d-alanine into teichoic acids [108,112]. GraSR was found to regulate the *dltABCD* and *mprF* genes [113,114]. In 2014, a loop region of sensor protein GraS was identified to recognize cationic peptides. Cheung *et al.* generated mutants of *graS* from the MRSA strain MW2. Deletion of *graS* (ΔgraS strain) or its 9-amino acid extracellular loop region (ΔEL strain) made the strain more susceptible to daptomycin, polymyxin B, human neutrophil defensin 1 (HNP-1), and RP-1 (a platelet factor 4 derived peptide that retains activity in blood). Meanwhile, these mutants became less infectious *in vivo* in an endocarditis model. Interestingly, a synthetic trimeric loop region EL mimic, *i.e.*, (DYDFPIDSL)_3_, could protect the parental MW2 strain from killing by those cationic peptides. These results suggest that the acidic residues in the extracellular loop region of GraS can directly interact with cationic peptides for sensing and activation [115].

It has been elucidated that Group A Streptococcus responds to the human antimicrobial peptide LL-37 by upregulating virulence factors controlled by the CsrRS system. In 2014, Velarde *et al.* identified RI-10, the smallest LL-37 fragment required to bind to CsrRS using a series of synthetic LL-37 fragments. RI-10 can directly bind to sensor kinase CsrS to activate the expression of virulence factors [116]. The same peptide was previously found by us to have no antibacterial activity against bacteria [97,98]. Since antimicrobial activity is not required for this recognition by kinase receptor, such a response could occur *in vivo* at a very low level of LL-37, which is not high enough to kill the bacteria. We propose that interfacial cationic residues R23 and K25, which are important for membrane permeation and bacterial killing [100], are also important residues for interaction with the acidic amino acids on the CsrS receptor.

#### 4.2.2. Gram-Negative Bacteria

Different from Gram-positive bacteria, LPS is the major component in the outer membranes of Gram-negative bacteria [66]. Modifications of bacterial LPS provide a general mechanism that confers resistance to cationic antimicrobial peptides [117]. The 2014 research on the genetic basis of bacterial resistance yielded additional support for this. First, pathogenic *Vibrio cholerae* strains can be >100-fold more resistant to polymyxins by modifying LPS (*i.e*., glycylation). Henderson *et al.* confirmed AlmF as an aminoacyl carrier protein, which is activated by the enzyme AlmE. Interestingly, these proteins in the AlmEFG trio system function in a manner similar to nonribosomal peptide synthetases [118]. Second, the resistance of human pathogen *Neisseria gonorrhoeae* results from phosphoethanolamine (PEA) decoration of lipid A by transferase encoded by the *lptA* gene [119,120,121]. Kandler *et al.* found that high-frequency mutation in a polynucleotide repeat of the *lptA* gene influences bacterial resistance. An *lptA* mutant is highly susceptible to cationic peptides [122]. In addition, the PEA-modification of lipid A has an immunostimulatory role during infection [123]. Third, in the case of the gastrointestinal pathogen *S. typhimurium*, resistance genes involving both LPS defects and mutation in *phoP* were found from a transposon library in 1992 [124]. The PhoPQ two-component system regulates peptide resistance, bacterial lipid A remodeling, and intracellular survival within acidified phagosomes. In 2014, the PhoPQ system was found to also regulate acidic glycerophospholipid content in the outer membrane [125]. These authors have recently summarized the resistance strategies of *S.* *typhi* [126,127].

In summary, both Gram-positive and Gram-negative bacteria are able to decorate their cellular surfaces to make them less attractive to cationic antimicrobial peptides. Interestingly, a recent study reveals that gut bacteria can use a similar mechanism by removing a phosphate group from LPS [128]. Here a resistant mechanism for “bad bugs” has become a survival strategy for “good bugs”. Therefore, such surface decorations achieved by a different chemistry provide a general mechanism that enables bacteria, for good or bad, to “work under an umbrella” to dodge the “bullets” of the host.

## 5. Potential Applications of Antimicrobial Peptides

### 5.1. Toward Therapeutic Uses

There is continued interest in developing therapeutic uses for antimicrobial peptides. Because of molecular simplicity and easy synthesis, linear peptides have been extensively explored for favorable properties. Here we highlight a structure-based design based on the human cathelicidin LL-37. Wang identified a chymotrypsin-resistant template by screening an LL-37 peptide library [129]. This template contains partial D-amino acids and has a novel amphipathic structure [130]. However, it is not active against community-associated methicillin-resistant *S. aureus* (MRSA) USA300. Based on the 3D structure, we enhanced anti-MRSA activity of the peptide by inserting a bulkier hydrophobic group into the structural cavity. One of the peptide analogs, 17BIPHE2, not only eliminated MRSA, but also recruited monocytes to the infection site [129]. In addition, other approaches were explored to make use of LL-37 or hBD-2. Since cathelicidin can reverse intestinal fibrosis in models of colitis [131], this peptide may be used to treat inflammatory bowel disease (IBD) by introducing cathelicidin-overexpressing bacteria. Using an adenoviral vector to deliver the gene of hBD-2, Woo *et al.* found both viral inhibition and clearance for experimental otitis media [132]. In 2014, LL-37-containing vector was electroporated to promote skin wound healing [133]. In addition, 1,25-dihydroxyvitamin D3 (active form) was also used to induce both LL-37 and hBD-2 production in keratinocytes from diabetic foot ulcers, promoting wound healing [134]. UV light or sunlight may be an alternative since hydroxylation of vitamin D can occur [135]. It is notable that traditional practice can also boost our immune systems. While yoga stretching significantly increases human hBD-2 [136], green tea helps the production of lactoferrin in saliva after exercise [137]. Although at the early stage, these positive results on antimicrobial peptides imply that our traditional life styles can be helpful to keep us healthy.

Unlike linear peptides that can be rapidly degraded by proteases in hours, circular peptides have inherent stability. This is because these peptides such as cyclotides comprise three conserved disulfide bonds in addition to a peptide bond that connects the N- and C-termini. Consequently, there is great interest in utilizing these natural templates to engineer useful therapeutics [138,139]. In 2014, Craik and colleagues demonstrated the molecular grafting technology where a desired antigenic peptide was inserted into an exposed loop region of kalata B1 [140]. This technology confers protease stability to the sequence motif, which can otherwise be rapidly degraded when tested alone. MOG3, one out of the seven peptides grafted to loop 5, was used to vaccinate mice and found to display *in vivo* efficacy in an animal model of multiple sclerosis, an inflammatory disease of the central nervous system. Taken together with previous examples [138,139], these authors proved the concept of segment grafting at loops 5 and 6 of cyclotides. It appears that sequence composition rather than length determines whether the grafted segment is tolerated without disrupting the protein fold. To obtain a sufficient amount of material for research, cyclotides were initially isolated from plants [141]. Later, chemical synthesis was established [142]. In 2014, two laboratories reported an alternative approach by using engineered sortase A to make the circular molecule [143,144]. A more efficient synthesis will bring us one step closer to practical use of these interesting templates.

### 5.2. Peptide Surface Coating

Immobilization of antimicrobial peptides (either covalently or adsorbed) onto solid supports extends their capabilities as surface-active molecules. This direction of research aims at improving biomedical devices, drug delivery systems, bio-sensor and detection, and so on. Recent advances in the field include simplification of the chemistry for surface attachment, development of novel substrate-attaching platforms, including nanomaterial for wider applications, product development with promising proof of concepts and *in vivo* testing in animal models. 

Of all the problems related to loss in efficiency of medically implantable medical devices is the development of microbial biofilms. Peptide immobilization has been shown to reduce bacterial colonization and biofilm formation. However, the major challenges that often hinder the immobilization of antimicrobial peptides are the inefficiency of the conjugation chemistries and their inability to achieve a sufficient surface concentration of peptides, along with the limited number of usable biomaterials. A recent study by Lim *et al.* [145] demonstrated a simple dopamine based chemical reaction for the functionalization of antimicrobial peptides onto a commonly used Silicon Foley catheter. Not only did the catheter prototype reduce biofilm formation by common pathogens that caused a urinary tract infection, it was also stable for 21 days. In addition, hLF1-11 immobilized onto titanium [146] has been shown to possess excellent anti-biofilm properties. There have also been improvements in other chemical platforms, including the development of thiolated self-assembled monolayer on a gold surface to which small peptides, temporin-SHf, can be tethered, resulting in broad-spectrum activity [147].

To understand the possible influence of structure and dynamics on immobilized antimicrobial peptides and to apply rational design, molecular dynamics simulation studies were carried out on cecropin P1 immobilized on silane-EG4-maleimide self-assembled monolayers [148]. Other factors governing immobilization reactions, such as spacer chain length, reactant concentration and energy dependence, are demonstrated by Mishra *et al.* [149]. Additional coating strategies can be found in a recent review [150]. Moving toward practical applications, a significant *in vivo* study is presented by Dutta *et al.* [151]. Melimine immobilized eye lenses are not only cytotoxically safe but also possess antibacterial activity after worn as tested in both rabbit and human. In addition, another peptide SESB2V immobilized on titanium surfaces prevents perioperative corneal infection in a rabbit keratitis model [152].

### 5.3. Nanoparticle-Based Drug Delivery Systems

Apart from the anti-biofilm and antibacterial functions, antimicrobial peptides tagged to nanoparticles impart a site-specific targeting and delivery of drug molecules. It can be used in treating a variety of diseases, including cancer. Currently, dermaseptin entrapped chitosan nanoparticles have been shown to possess excellent antitumor activities [153]. Moving one step forward, dual targeting nanoparticles with both blood−brain barrier (BBB) and blood−brain tumor barrier (BBTB) including glioma cell were achieved by functionalizing lactoferrin to the surface of poly(ethylene glycol)−poly(lactic acid) nanoparticles. Administration with tLyP-1, a tumor-homing peptide that mediates tissue penetration through the neuropilin-1-dependent internalization pathway, resulted in deep penetration of the nanoparticle into the glioma parenchyma [154]. It opens a new direction for administration of antitumerogenic drugs with high penetration capability.

Additionally, glutamic acid substitution of basic residues in LL-37, melittin, and bombolitin V linked to lipid nanoparticles could be used for endosomal escape and efficient gene delivery using intravenous injections. These yield expression levels comparable to those obtained using Lipofectamine 2000 and the probable mode of action resembles viruses [155]. Antimicrobial peptides have also been shown to have superb drug releasing properties in PEG-PLGA microparticles [156]. The direct evidence is presented by encapsulating LL-37 in PLGA nanoparticles by Chereddy *et al.* [157]. PLGA-LL-37 nanoparticles as a biodegradable drug delivery system were found to promote wound closure. It functions due to the sustained release of both LL-37 and lactate, and induction of enhanced cell migration without effects on the metabolism and proliferation of keratinocytes. Moreover, peptides as short as dimer conjugated to naphthalene could be used as antimicrobial nanomaterials in eliminating biofilm infections and for drug delivery [158].

### 5.4. Biosensors and Detection

Antimicrobial peptides can also serve as indicators and diagnostic agents. The approach is more cost effective than standard PCR or antibody-based techniques. Detection of bacterial pathogens in a microfluidic chip where antimicrobial peptides are immobilized via cysteine-gold interaction could produce a rapid electrical detection with sufficient sensor signal that allows the detection of pathogens (both Gram-negative and Gram-positive) at a density as low as 10^5^ cfu/mL within 25 min [159]. While another platform for the detection of only Gram-positive bacterial strains could reach 10^3^ cfu/mL via immobilizing class IIa bacteriocins [160]. In addition, specific detection of fungal *C. albicans* can also be made by using peptide nucleic acid probes [161].

## 6. Perspectives

It is great news that acquired resistance did not become an issue after decade-long use of antimicrobial peptides such as tyrothricin [162]. Such an observation is encouraging to the current effort in development of antimicrobial peptides into novel therapeutic molecules [63]. From 2000 to 2014, about 100 new antimicrobial peptides were entered into the APD every year [26]. As of December 2014, there were 2493 peptides in this database [7,8,9]. We anticipate that the important work on the isolation and characterization of novel antimicrobial peptides from new organisms will continue. Scientifically, new peptide sequences will improve our knowledge of natural antimicrobial peptides. As shown in Table 1, the new members can refine the boundary parameters for natural antimicrobial peptides [9]. With the identification of a sufficient number of representative peptide sequences, the APD database will more accurately identify natural peptides most similar to the input sequence. It will facilitate the development of new programs to better predict the likelihood of a new peptide to be antimicrobial. In addition, a unique peptide may directly become a candidate lead for the development of novel antimicrobials to meet the challenge of the antibiotic resistance problem. However, it is important to validate whether a bacteriocin has any undesired virulent property that promotes infection [21].

The 2014 research also enabled us to connect the dots for known human antimicrobial peptides, leading to an improved understanding of their functional roles in innate immunity. Surprisingly, a different form of human cathelicidin peptide can be rapidly expressed by skin fat cells in response to the *S. aureus* invasion, underscoring a direct defense role of human cathelicidin peptides [50]. It is remarkable that a single LL-37 molecule can perform multiple functions by recognizing a variety of molecular partners or receptors on immune cells. In 2014, pH-dependent oligomerization of LL-37 is connected to the delivery of dsRNA into endosomes for subsequent interactions with TLR3 [64]. Although the details are to be elucidated, we propose that the terminal regions of LL-37 contain an important molecular switch based on NMR data [63]. It is demonstrated that local conditions are essential for a proper function of human antimicrobial peptides. While human HD-6, like hBD-1, is active only after disruption of disulfide bonds under reduced conditions [43], many other defensins in the folded form can directly recognize specific lipids in pathogen’s membranes [163,164]. During such a molecular recognition process, the flexible residues in the loop regions of these small defense proteins are found to be essential based on several structural studies [163,164,165,166].

The 2014 results further expanded our view on the mechanism of action of antimicrobial peptides (summarized in Figure 1). While select antimicrobial peptides are known to inhibit cell wall synthesis, many target bacterial membranes. Surface-binding peptides are shown to be able to replace weakly attached membrane proteins, thereby interfering with bacterial physiology globally [94]. Beyond membranes, bacteriocins can use cell receptors as a target [81]. It has been known for a while that proline-rich peptides interact with heat shock proteins [92]. However, the molecular target has now been traced to ribosomal proteins. The binding of proline-rich peptides leads to inhibition of protein synthesis [84,85].

Although there are few examples, structure-based design has been demonstrated [129,140], leading to antimicrobials or vaccines with desired properties. The overall goal of peptide engineering is to establish or identify a proper peptide template with desired potency, stability, and cell selectivity. In addition, one may also mimic nature’s wisdom. Based on the precursor protein construction, one can design pro-peptide forms to minimize potential cytotoxicity and protease degradation. In 2014, Forde *et al.* illustrated this strategy as a potential therapy for cystic fibrosis [167,168]. Nature has created other strategies to circumvent rapid peptide degradation by forming a complex structure. For instance, human LL-37 can oligomerize at physiological pH into nanoparticles to resist the action of proteases [63]. Likewise, LL-37 can bind to DNA to stabilize the entire NETs structure [169]. In 2014, Bachrach and colleagues showed that human LL-37 could also be protected by actin, thereby maintaining its antimicrobial activity *in vivo* [170,171]. There are also natural ways to reduce potential cytotoxicity of antimicrobial peptides. In 2014, Svensson *et al.* discovered that peptide p33 expressed on the surface of various cell types can reduce the potential cytotoxicity of human LL-37 [172]. Moreover, Hiemstra *et al.* discovered the nanoparticle-like vesicles in the human urinary tract [173]. It is predictable that novel functional modes of human innate immune peptides will continue to emerge. All these natural mechanisms may hold the key to future therapeutics.

We anticipate continued efforts in the development of potential applications of antimicrobial peptides, including peptide production methods. Peptide engineering, formulation, and delivery technologies may further expand the horizon of antimicrobial peptides in benefiting human beings [174]. Such applications can vary from medical surface cleaning, water quality monitoring and disinfection, sterile surface materials, to new drugs for infectious diseases [175,176]. Antimicrobial peptides may be included in existing detergent formulation and disinfectants to reduce bacterial biofilms on hospital surfaces [177]. Bovicin HC5 and nisin can be used to treat food-contact surface to reduce bacterial attachment and subsequent biofilm formation [178,179]. It has been demonstrated that injection of an engineered LL-37 peptide or coating peptides to the device surface can prevent biofilm formation [129,145,146]. Importantly, a combined use of antimicrobial peptides with traditional antibiotics can be a more effective strategy to treat bacterial biofilms [180,181,182]. Finally, molecules that interfere with or even weaken the process of biofilm formation [183,184,185,186,187,188,189] can also be combined with antimicrobial peptides to achieve an optimal treatment of bacterial biofilms.
